# Utilization of Ligasure^®^ Maryland Jaw Open Sealer/Divider with Nanocoating Improves Perioperative Parameters in Women with Advanced Ovarian Cancer Subjected to Cytoreductive Surgery

**DOI:** 10.3390/jcm14176293

**Published:** 2025-09-05

**Authors:** Dimitrios Tsolakidis, Kimon Chatzistamatiou, Efthalia Markopoulou, Dimitrios Zouzoulas, Vasilis Theodoulidis, Panagiotis Tzitzis, Iliana Sofianou, Kalliopi Kissoudi, Maria Topalidou, Eleni Timotheadou, Grigorios Grimbizis

**Affiliations:** 1 1st Department of Obstetrics and Gynaecology, Papageorgiou General Hospital, Aristotle University of Thessaloniki, 56403 Thessaloniki, Greece; dtgyn@auth.gr (D.T.); efthaliamarkopoulou@gmail.com (E.M.); dzouzoulas@hotmail.gr (D.Z.); theodoulidisvasilis@yahoo.gr (V.T.); ptzitzis@gmail.com (P.T.); iliana.m.sofianou@gmail.com (I.S.); kissoudi.c@gmail.com (K.K.); grigoris.grimbizis@gmail.com (G.G.); 2 Department of Radiation Oncology, Papageorgiou General Hospital, 56403 Thessaloniki, Greece; mariatop@gmail.com; 3 Department of Medical Oncology, Papageorgiou General Hospital, 56403 Thessaloniki, Greece; timotheadou@gmail.com

**Keywords:** ovarian cancer, cytoreductive surgery, ligasure, perioperative parameters

## Abstract

**Background/Objectives**: Cytoreductive surgery for women with advanced ovarian cancer is a demanding process with high morbidity. The present analysis aims to identify whether using the Ligasure^®^ Maryland jaw open sealer/divider (LMJsd) with a nanocoating (Covidien^®^, Medtronic^®^, 710 Medtronic Parkway, Minneapolis, MN, USA), could lead to better outcomes during cytoreduction surgery by reducing intraoperative bleeding and other hospitalization-related parameters. **Methods**: Patients with ovarian cancer (FIGO III/IV) who were subjected to primary or interval cytoreductive surgery at the Gynecologic-Oncology Unit, 1st Department of Obstetrics and Gynecology, Papageorgiou General Hospital, Thessaloniki, Greece, were included in the analysis. Patients were retrospectively allocated into two groups: women operated on with or without using the LMJsd. Differences between the two groups (intraoperative blood loss and blood transfusion, duration of surgery, postoperative blood transfusion, admission to intensive care unit (ICU), and overall hospital length of stay) were investigated. **Results**: From 2012 to 2020, 284 women with ovarian cancer were surgically treated; 208 were stage III/IV. In the LMJsd group of women (n = 34), the duration of surgery and blood loss during surgery were significantly decreased (*p* < 0.0005) compared to the non-LMJsd group (n = 174). The intraoperative blood transfusion rate and the number of packed red blood cell units transfused were significantly decreased in the first group (*p* = 0.0025); the postoperative blood transfusion rate was not different (*p* = 0.065). Moreover, ICU admission and overall hospital length of stay were significantly decreased in the LMJsd group (*p* < 0.0005 and *p* = 0.015). **Conclusions**: Using the LMJsd is associated with decreased intraoperative bleeding and transfusion rates, duration of surgery, admission to ICU, and overall hospital length of stay in women treated with surgical cytoreduction for advanced ovarian cancer. Some limitations of this study are as follows: its limited impact because it is an observational retrospective analysis and bias because the cumulative experience of the surgeons may have an impact on the surgical outcomes.

## 1. Introduction

Ovarian cancer is the eighth most common cancer in women, with an incidence of 6.6 new cases per 100,000 women per year. In 2020, there were more than 313,000 women who were newly diagnosed with ovarian cancer and more than 207,000 women who died from the disease worldwide (GLOBOCAN 2020, www.gco.iarc.fr, URL accessed on 20 November 2024). Moreover, the secondary prevention of ovarian cancer has not been proven effective to date in terms of reducing ovarian cancer deaths [1]. Thus, the effective treatment of ovarian cancer still remains the main focus of disease control.

Cytoreductive surgery is the cornerstone treatment in the armamentarium for women with advanced ovarian cancer. It has been shown that maximal cytoreduction is the strongest predictor factor of survival among women with advanced-stage ovarian cancer [2]. The goal of successful cytoreduction is to achieve no visible tumor or residual disease less than 1 cm. This prerequisite necessitates a demanding surgical process with high morbidity, requiring great clinical expertise and enhanced surgical skills. This process is also described by the term “ultraradical surgery”, and in recent years, there has been significant progress in the dissemination of several techniques required for this surgical approach such as the “Hudson” procedure with en block resection of anatomical structures within the pelvis, diaphragmatic stripping or resection after liver mobilization, and resection of supradiaphragmatic or cardiophrenic lymph nodes. For this reason, there are specialized centers practicing and teaching “ultraradical” surgery by using the technological advancements that have made extensive surgery faster, bloodless, feasible, and safe [3,4,5,6]. One of these technological advancements is the Ligasure^®^ Maryland jaw open sealer/divider (LMJsd) with a nanocoating [7].

The objective of the present analysis is to investigate whether the usage of the LMJsd facilitates cytoreductive surgery by reducing intraoperative bleeding and hence positively affecting other parameters associated directly or indirectly with morbidity.

## 2. Materials and Methods

### 2.1. Study Design

The present analysis is a retrospective unmatched case–control study on the utilization of the LMJsd in the surgical treatment of women with advanced ovarian cancer, who were referred to the Unit of Gynaecological Oncology, 1st Department of Obstetrics and Gynecology, Papageorgiou General Hospital, Thessaloniki, Greece. This unit has been recognized as an accredited European training center for gynecological oncology, as well as an accredited center for advanced ovarian cancer surgery by the European Society of Gynaecological Oncology (ESGO).

The patient records of women with advanced ovarian cancer (stages III/IV) were retrieved. These women were subjected to either primary or interval cytoreductive surgery according to a multidisciplinary tumor (MDT) board decision, and they were retrospectively allocated to two distinct groups, the LMJ group and the non-LMJ group, depending on whether the LMJsd was used or not.

To identify possible effects regarding the usage of the LMJsd on the course of cytoreductive surgery, we investigated differences between certain parameters related to the intraoperative or postoperative course of the patients, between the two groups. Intraoperative parameters were as follows: intraoperative blood loss; duration of surgery; residual disease, defined as all visible tumor after the completion of surgery; intraoperative transfusion rate; and units of packed red blood cells (pRBCs) transfused. Postoperative parameters were as follows: length of stay in the intensive care unit (ICU), total length of stay in the hospital, postoperative blood transfusion rate, number of units of pRBCs transfused, and the difference between preoperative and the lowest intra- or postoperative serum hemoglobulin levels (Hb difference).

### 2.2. Surgical Procedures

All the participants were subjected to either primary or interval cytoreductive surgery. Patient selection depended primarily on the operability of the disease and was assessed either with diagnostic laparoscopy or according to preoperative imaging modalities. The goal of the procedure was complete cytoreduction in both instances. All the procedures were conducted with laparotomy (midline vertical incision), including but not being limited to the following elements: total hysterectomy with bilateral salpingo-oophorectomy, removal of enlarged pelvic and/or para-aortic lymph nodes, radical supracolic omentectomy, splenectomy, cholecystectomy, appendicectomy, colorectal and/or small bowel resection with anastomosis, pelvic peritonectomy, anterior parietal or paracolic gutter peritonectomy, diaphragmatic stripping, and the Hudson procedure.

The surgical technique included sharp or blunt dissection, cutting, and ligating according to the basic principles of abdominal surgery. For the non-LMJ group, this was performed using monopolar and/or bipolar diathermy by conventional electrosurgical instruments. For the group treated with the LMJsd, all basic surgical maneuvers and resections were performed using this instrument, except pelvic peritonectomy, anterior parietal peritonectomy, and diaphragmatic stripping, which were performed using monopolar ball diathermy (3 mm in diameter). The usage of the LMJsd was based upon device availability and surgeon preference during specific operative sessions, rather than patient-specific factors.

The LMJsd is a vessel sealing device, designed and produced by Covidien^®^, Medtronic^®^ 710 Medtronic Parkway, Minneapolis, MN, USA. It combines the Ligasure technology, creating optimal conditions to achieve consistent sealing, with the known Maryland jaw, characterized by multi-functionality, including blunt dissection, atraumatic tissue grasping, and cold cutting when necessary. Another feature of this device is its nanocoating which can reduce eschar buildup and decrease instances of sticking, requiring fewer cleanings of the jaw [7].

### 2.3. Statistical Analysis

Descriptive statistics (mean, standard deviation, median, range) were used to present the demographic characteristics of the two groups included in the analysis, and an independent samples *t*-test or Pearson chi-square test was used to assess statistical differences between the two groups, regarding demographic parameters.

Intraoperative and postoperative parameters were investigated to assess differences between the LMJ and non-LMJ groups, comprising either continuous or categorical variables. Continuous variables were the following: intraoperative blood loss, measured in milliliters (mL); duration of surgery, measured in minutes (min); duration of ICU stay; duration of overall hospital stay, measured in days; and the Hb difference, measured in milligrams per deciliter (mg/dL). Categorical variables were as follows: residual disease (ordinal variable), a variable with three values, namely zero, when no residual disease was visible after the completion of surgery, and <1 cm and >1 cm, when the maximum diameter of residual disease, isolated or multifocal, was less than or more than 1 cm, respectively; ICU stay, a variable which defined whether a patient was admitted to the ICU or not, irrespective of the ICU length of stay; intraoperative and postoperative blood transfusion, two variables defining the occurrence of blood transfusion or not irrespective of the number of pRBCs being transfused; and pRBCs, an ordinal variable defining the number of units of pRBCs transfused.

Differences between the two groups of women participating in this study, regarding continuous variables, were statistically assessed using an independent samples *t*-test. Differences regarding categorical variables were assessed using the Pearson chi-square test, and ordinal variables, namely residual disease and the number of pRBCs transfused, were assessed using the Mann–Whitney U-test. Moreover, multivariate analyses were performed using a Multivariate Analysis of Covariance (MANCOVA), and differences were assessed with an F-test. *p*-values of less than 0.05 defined statistical significance. All analyses were performed with SPSS^®^ statistical software, 25th edition (IBM^®^, 1 New Orchard Road, Armonk, NY, USA).

## 3. Results

### 3.1. Baseline Characteristics

Between 2012 and 2020, 284 women with ovarian cancer were subjected to surgery; of these, 208 had ovarian cancer stage III or IV. These women were subjected to cytoreductive surgery with (n = 34, LMJ group) or without (n = 174, non-LMJ group) the use of the LMJsd.

Table 1 describes the baseline patient characteristics. The mean age was 55.9 and 60.7 years for the LMJ and non-LMJ groups, respectively (*p* = 0.047). All women recruited for this analysis were Caucasian, and most of them were post-menopausal: 73.5% in the LMJ group and 77.6% in the non-LMJ group (*p* = 0.61). No statistically significant differences between the two groups were found regarding Body Mass Index (BMI), American Society of Anesthesiologists physical status score (ASA score), or the main aspects of the past medical history of the women recruited (Table 1).

Most women were diagnosed with stage III ovarian cancer: 8 (2.9%) women were diagnosed with stage IIIa, 18 (8.7%) with stage IIIb, and 160 (76.9%) with stage IIIc. There were 22 women diagnosed with stage IV ovarian cancer: 9 (4.3%) women were diagnosed with stage IVa and 13 (6.3%) women with stage IVb (Table 2). The mean Ca-125 levels at diagnosis were similar between the two groups of women (1227.3 and 1024.8 for the LMJ and non-LMJ groups, respectively). Regarding the histological types of ovarian cancer, 83.6% of the total study population were serous-papillary, 7.7% were endometrioid, 2.4% were mucinous, 2.4% were clear-cell, and 3.8% were other subtypes (sex-cord, undifferentiated, etc.). The serous-papillary ovarian cancer histologic type was slightly less frequently identified in the LMJ group (73.5%) compared to the non-LMJ group (85.5%) (*p* = 0.011) (Table 2).

### 3.2. Perioperative Surgical Parameters

Table 3 presents the different surgical procedures performed in the framework of cytoreductive ultraradical surgery in the two groups of women included in the analysis. The only differences with a statistical significance were reported in radical hysterectomy (performed in six cases in the LMJ group and in no case in the non-LMJ group, *p* < 0.001), in pelvic peritonectomy (performed in 73.5% in the LMJ group and in 54.6% in the non-LMJ group, *p* = 0.041), and in anterior parietal peritonectomy (performed in 17.6% in the LMJ group and in 6.3% in the non-LMJ group, *p* = 0.039). In all the remaining procedures, no statistically significant differences were observed (Table 3). Overall, the surgical complexity score (SCS) [8] was not statistically significantly different between the two groups.

In the group of women who were operated on using the LMJsd, blood loss during surgery was significantly reduced (*p* < 0.001) compared to cases treated without the LMJsd. The mean intraoperative blood loss for the total population was 590 mL (417.7 mL for the LMJ group and 625.3 for the non-LMJ group). The intraoperative blood transfusion rate was significantly lower in the LMJ group (*p* = 0.029), whereas the postoperative blood transfusion rate was not affected (*p* = 0.171). Overall, 52.4% of the patients required intraoperative blood transfusion. This rate was 55.7% for the non-LMJ group and 39.3% for the LMJ group (*p* = 0.029). Regarding postoperative blood transfusion, although the rates were higher for the LMJ group (55.9% compared to 43.1% for the non-LMJ group), this did not reach statistical significance (*p* = 0.171). Moreover, in the case of a transfusion, the number of packed red blood cells given intraoperatively or postoperatively did not significantly differ between the two groups of women (*p* = 0.748 and *p* = 0.743, respectively) (Table 4). It should also be noted that the Clavien–Dindo [9] index for the classification of complications in patients subjected to surgical operations was not different between the two groups.

Finally, Table 4 presents the effects of surgical complexity on perioperative outcomes. It is shown that between the two groups of women, in the case of a high surgical complexity score, intraoperative blood loss and hospital stay were not statistically different (*p* = 0.122 and 0.207, respectively). Also, in multivariate analysis, it is shown that, after controlling for surgical complexity, residual disease, age, and histologic type, blood loss and duration of surgery still retain statistical significance (*p* < 0.020 and *p* < 0.001, respectively), but the overall hospital length of stay does not (*p* = 0.24).

Women who were operated on with the LMJsd were more likely to be transferred to the intensive care unit (ICU) immediately after the operation (*p* < 0.001); 64.7% of the women allocated to the LMJ group were transferred to the ICU compared to 22.5% of the women in the non-LMJ group. This fact, however, probably reflects a change in postoperative management in recent years in our center. Furthermore, when ICU transfer was decided, the duration of the stay in the ICU was shorter for the LMJ grsoup compared to the non-LMJ group (1.3 vs. 2.7 days; *p* < 0.001). The overall hospital length of stay was also significantly reduced in cases where the LMJsd was used (7.8 days for the LMJ group compared to 8.9 days for the non-LMJ group, *p* = 0.029) (Table 4).

The surgical outcome, in terms of successful cytoreduction, was similar between the two groups. Complete or adequate cytoreduction, defined as residual disease of less than 1 cm, was achieved in 85.3% of cases in the LMJ group and in 88.5% of cases in the non-LMJ group (*p* = 0.314). The duration of surgery, however, depended on the usage of the LMJsd; surgery in the LMJ group had a mean duration of 313.5 min, whereas in the non-LMJ group, the mean duration was 231.5 min (*p* < 0.001) (Table 4).

## 4. Discussion

Ovarian cancer is a great cancer-related burden in women, and it has a five-year survival rate of less than 45%. It still represents a significant cause of morbidity and mortality for women worldwide, and its incidence has been increasing in both high-income and many low- and middle-income countries because of the aging of the population [10]. In recent years and due to the significant effects of ovarian cancer on public health, efforts have been made in order to identify the utility of population-based screening for the early diagnosis or even prevention of ovarian cancer [11]. The most recent randomized controlled trial investigating ovarian cancer screening was the UK Collaborative Trial of Ovarian Cancer Screening (UKCTOCS). This trial investigated annual multimodal screening (MMS), which included CA125 testing followed by repeat CA125 and transvaginal ultrasound (TVS) as a second-line test, TVS alone, or no screening. Although some ovarian cancers were detected earlier with MMS, this was not sufficient to improve the prognosis of those cancers and did not translate into a reduction in mortality from the disease [1]. This study made it clear that ovarian cancer prevention is not possible using the current biomarkers or imaging modalities, a fact that enhances the significance of effective treatment in order to control the disease. The cornerstone of ovarian cancer treatment is the surgical removal of all visible tumor, resulting in zero residual disease, achieved in the last decade by the introduction of ultraradical surgery. However, this type of surgery is associated with adverse perioperative outcomes linked, among other things, to intraoperative hemorrhage.

There have been technological advancements in energy-based technologies and various instruments aiming to reduce intraoperative hemorrhage, which have been implemented in ultraradical abdominal surgery. In our center we have been using the LMJsd since January 2019, for either primary or interval cytoreductive surgery in advanced ovarian cancer cases. The current study aimed to evaluate the impact of using the LMJsd on various perioperative outcomes including blood loss, transfusion rates, ICU transfer, length of hospital or ICU stay, surgical outcomes, and duration of surgery. The results indicated several significant findings that contribute to our understanding of the benefits of using the LMJsd. One notable finding was the significant reduction in blood loss during surgery among women operated on using the LMJsd compared to those treated without it. This finding is consistent with previous research that has demonstrated the effectiveness of sealing devices in minimizing intraoperative blood loss during various procedures compared to usual practice [12,13,14]. The reduction in blood loss during surgery is a critical parameter affecting the quality of the surgical procedure performed as well as the postoperative course of the patient. In line with our results, Ligasure^®^ devices have been shown to be effective in reducing blood loss in a variety of operations in gynecological oncology including but not limited to radical hysterectomy, pelvic and para-aortic lymphadenectomy, and omentectomy [15,16].

In line with the reduced blood loss, our study also found a significantly lower intraoperative blood transfusion rate in the group of women treated with the LMJsd, suggesting decreased reliance on blood transfusions during surgery, which is not only beneficial for patients but also helps to optimize healthcare resources. Interestingly, although there was a higher postoperative blood transfusion rate in the LMJ group, this difference did not reach statistical significance, and it could be attributed to various factors, such as patient characteristics, surgical techniques, or variations in postoperative care; nonetheless, overall transfusion rates were lower in women treated with the LMJsd.

Another significant finding is the higher rate of ICU transfer immediately after surgery among women in the LMJ group. We were not able to identify possible associations explaining this finding, but we think that the longer duration of surgery in the LMJsd group might have played a role. Nonetheless, the shorter duration of ICU stay for the LMJ group may suggest improved postoperative recovery, indicating that the use of the LMJsd could potentially contribute to enhanced patient outcomes and resource utilization.

Moreover, a significant reduction in the overall hospital length of stay was observed when the LMJsd was utilized. A reduced hospital length of stay is one of the strategies that has been used to ensure healthcare systems’ sustainability and one of the key performance indicators, as it is widely used to assess hospital efficiency. Also, from the individual patient perspective, a prolonged length of stay may impose a negative effect on the patient’s health status due to an increased risk of a hospital-acquired infection, such as urinary and respiratory tract infections and other complications [17].

A negative outcome of the use of the LMJsd is the increased duration of the surgical operation; this fact, visualized through the prism of the abovementioned improved perioperative outcomes, is not considered a significant drawback for the utilization of the instrument, also given the fact that the mean increase in duration was only about 80 min.

This study has some limitations. First, it is an observational retrospective analysis, and as such the results cannot be universally adopted. Second, there is bias coming from the fact that the non-LMJ group was mainly treated in earlier years compared to the LMJ group; therefore, the cumulative experience of the surgeons may have impacted the surgical outcomes in favor of the LMJsd group. However, since this study presents the results of a single institution and a single group of surgeons/anesthetists/nurses, the same processes were followed regarding perioperative treatment for both groups. Also, the size of the intervention group is significantly smaller than that of the control group; despite this fact, consistent trends were observed across multiple endpoints. Another limitation is the fact that the LMJsd was chosen depending on the surgeons’ preference or instrument availability. Finally, due to the exploratory and observational nature of this study, no power analysis was performed.

## 5. Conclusions

In conclusion, the findings of this study highlight the potential benefits of using the LMJsd during cytoreductive surgery for advanced ovarian cancer. The LMJsd may contribute to reducing blood loss, transfusion rates, ICU stay, and overall hospital length of stay while maintaining comparable, optimal surgical outcomes. Further research is warranted to validate these findings prospectively in larger cohorts and different surgical settings.

## Figures and Tables

**Table 1:** The baseline patient characteristics of the two groups of women with ovarian cancer included in the analysis.

	Total Study Population	LMJ Group	Non-LMJ Group	*p*-Value
	N = 208	n = 34	n = 174	
Age (years)				
Mean (Sd)	59.9 (12.8)	55.9 (14.6)	60.7 (12.3)	0.047 *
Median (range)	60 (17–86)	55.5 (17–81)	61.5 (21–86)	
Ethnic origin				
White Caucasian	208 (100%) ^$^	34 (100%) ^$$^	174 (100%) ^$$$^	
Post-menopausal	160 (76.9%) ^$^	25 (73.5%) ^$$^	135 (77.6%) ^$$$^	0.61 *
Number of children				0.63 **
None	33 (15.8%) ^$^	8 (23.5%) ^$$^	25 (14.4%) ^$$$^	
1–2	141 (67.8%) ^$^	18 (52.9%) ^$$^	123 (70.7%) ^$$$^	
>3	28 (13.5%) ^$^	7 (20.6%) ^$$^	21 (12.1%) ^$$$^	
ASA score				
1	37 (23.6%) ^$^	11 (34.4%) ^$$^	26 (20.8%) ^$$$^	0.158 **
2	101 (64.3%) ^$^	16 (50.0%) ^$$^	85 (68%) ^$$$^	
3	19 (12.1%) ^$^	5 (15.6%) ^$$^	14 (11.2%) ^$$$^	
BMI				
Mean (Sd)	30.3 (20.3)	30.2 (5.7)	28.7 (4.08)	0.181 *
Median (range)	28.5 (17–86)	29.6 (20.3–42.8)	28.5 (17.2–47.3)	
Past medical history				
Diabetes	15 (7.2%) ^$^	6 (17.6%) ^$$^	9 (5.2%) ^$$$^	0.1 **
Hypertension	58 (27.9%) ^$^	8 (23.5%) ^$$^	50 (28.7%) ^$$$^	0.536 **
Tobacco use	51 (24.5%) ^$^	5 (9.8%) ^$$^	46 (26.4%) ^$$$^	0.146 **
Vascular disease	12 (5.8%) ^$^	5 (14.7%) ^$$^	7 (4.0%) ^$$$^	0.15 **

LMJ: Ligasure Maryland-jaw; ASA: American Society of Anesthesiologists; Sd: standard deviation; BMI: Body Mass Index. ^$^ percentages calculated within total study population; ^$$^ percentages calculated within LMJ group; ^$$$^ percentages calculated within non-LMJ group. * *p*-value from independent samples *t*-test; ** *p*-value from Pearson chi-square test.

**Table 2:** The disease characteristics of the two groups of women with ovarian cancer included in the analysis.

	Total Study Population	LMJ Group	Non-LMJ Group	*p*-Value
FIGO stage				
IIIa	8 (2.9%) ^$^	2 (5.9%) ^$$^	6 (3.4%) ^$$$^	0.112 *
IIIb	18 (8.7%) ^$^	0 (0.0%) ^$$^	18 (10.3%) ^$$$^	
IIIc	160 (76.9%) ^$^	31 (91.2%) ^$$^	129 (74.1%) ^$$$^	
IVa	9 (4.3%) ^$^	0 (0.0%) ^$$^	9 (5.2%) ^$$$^	
IVb	13 (6.3%) ^$^	1 (7.7%) ^$$^	12 (6.9%) ^$$$^	
CA-125 (mean, sd) (mg/dL)	1057.1 (2402.4)	1227.3 (2106.8)	1024.8 (2459.1)	0.668 **
Histologic type				
Serous-papillary	173 (83.6%)	25 (73.5%)	148 (85.5%)	0.011 *
Endometrioid	16 (7.7%)	5 (14.7%)	11 (6.4%)	
Mucinous	5 (2.4%)	0 (0.0%)	5 (2.9%)	
Clear-cell	5 (2.4%)	0 (0.0%)	5 (2.9%)	
Other subtypes	8 (3.8%)	4 (11.7%)	4 (2.3%)	
Grade				
1	6 (2.9%)	1 (3.1%)	5 (2.9%)	0.987 *
2	60 (29.3%)	9 (28.1%)	51 (29.5%)	
3	139 (67.8%)	22 (68.8%)	117 (67.6%)	

LMJ: Ligasure Maryland Jaw; FIGO: International Federation of Gynaecology and Obstetrics; sd: standard deviation. ^$^ percentages calculated within total study population; ^$$^ percentages calculated within LMJ group; ^$$$^ percentages calculated within non-LMJ group. * *p*-value from chi-square test; ** *p*-value from independent samples *t*-test.

**Table 3:** The surgery characteristics of the two groups of women with ovarian cancer included in the analysis.

	Study Population	LMJ Group	Non-LMJ Group	*p*-Value
TAH +/− BSO	185 (88.9%)	28 (82.4%)	157 (84.9%)	0.18
Radical hysterectomy	6 (2.9%)	6 (17.6%)	0 (0.0%)	<0.001
Pelvic lymphadenectomy	47 (22.6%)	10 (29.4%)	37 (21.3%)	0.299
Para-aortic lymphadenectomy	48 (23.1%)	9 (26.5%)	39 (22.4%)	0.608
Radical omentectomy	195 (93.8%)	32 (94.1%)	163 (93.7%)	0.923
Splenectomy	13 (6.3%)	1 (2.9%)	12 (6.0%)	0.384
Cholecystectomy	5 (2.4%)	2 (5.9%)	3 (1.7%)	0.148
Appendicectomy	41 (19.7%)	7 (20.6%)	34 (16.3%)	0.888
Colorectal resection	41 (19.7%)	7 (20.6%)	34 (19.5%)	0.888
Small bowel resection	8 (3.8%)	2 (5.9%)	6 (3.4%)	0.5
Pelvic peritonectomy	120 (57.7%)	25 (73.5%)	95 (54.6%)	0.041
Anterior parietal peritonectomy	17 (8.2%)	6 (17.6%)	11 (6.3%)	0.039
Diaphragmatic stripping	35 (16.8%)	7 (20.6%)	28 (16.1%)	0.522
SCS				
SCS (mean, sd)	4.92 (2.388)	5.15 (2.148)	4.87 (2.436)	0.539
Low SCS (≤3)	66 (31.7%)	7 (20.6%)	59 (33.9%)	0.112
Intermediate SCS (4–7)	115 (55.3%)	21 (61.8%)	94 (54%)	
High SCS (≥8)	27 (13%)	6 (17.6%)	21 (12.1%)	

TAH: total abdominal hysterectomy; BSO: bilateral salpingo-oophorectomy; LMJ: Ligasure Maryland Jaw; SCS: surgical complexity score.

**Table 4:** A comparison of different parameters between the two groups of women with ovarian cancer included in the analysis.

	Total Study Population	LMJ Group	Non-LMJ Group	Statistical Test (*p*)	Low SCS	Intermediate SCS	High SCS	Multivariate Analysis #
Intraoperative blood loss [mean (sd) (mL)]	590 (519.9)	414.7 (214.1)	625.3 (554.4)	<0.001 *	0.037 *	0.002 *	0.122 *	<0.02
Duration of surgery [mean (sd) (min)]	245 (90.7)	313.5 (77.2)	231.5 (87.2)	<0.001 *	0.003 *	0.001 *	0.028 *	<0.001
Rd								
0	139 (66.8%) ^$^	26 (76.5%) ^$$^	113 (64.9%) ^$$$^	0.314 ***	0.342 ***	0.5 ***	0.916 ***	-
<1 cm	44 (21.2%) ^$^	3 (8.8%) ^$$^	41 (23.6%) ^$$$^					
>1 cm	25 (12.0%) ^$^	5 (14.7%) ^$$$^	20 (11.5%) ^$$$^					
ICU stay								
No [n (%)]	148 (71.1%) ^$^	12 (35.3%) ^$$^	136 (77.5%) ^$$$^	<0.001 **	<0.001 **	0.017 **	0.007 **	-
Yes [n (%)]	60 (28.9%) ^$^	22 (64.7%) ^$$^	38 (22.5%) ^$$$^					
ICU stay duration [mean (sd) (days)]	2.1 (1.6)	1.3 (0.6)	2.7 (1.9)	<0.001 *	0.178 *	0.013 *	0.041 *	-
Hospital stay [mean (sd) (days)]	8.7 (5.3)	7.8 (1.2)	8.9 (5.8)	0.029 *	0.914 *	0.011 *	0.207 *	0.24
Intraoperative transfusion								
No [n (%)]	99 (47.6%) ^$^	22 (64.7%) ^$$^	77 (44.3%) ^$$$^	0.029 **	0.165 ***	0.739 **	<0.001 **	-
Yes [n (%)]	109 (52.4%) ^$^	22 (39.3%) ^$$^	97 (55.7%) ^$$$^					
pRBC units				0.748 ***	0.761 ***	0.602 ***	0.003 ***	-
Postoperative transfusion								
No [n (%)]	114 (54.8%) ^$^	15 (44.1%) ^$$^	99 (56.9%) ^$$$^	0.171 **	0.429 **	0.344 **	0.707 **	-
Yes [n (%)]	94 (45.2%) ^$^	19 (55.9%) ^$$^	75 (43.1%) ^$$$^					
pRBC units				0.743 ***	0.761 ***	0.987 ***	0.760 ***	-
Preoperative Hb (mean, sd) (mg/dL)	12.0 (1.48)	11.8 (1.22)	12.07 (1.52)	0.291 *	0.508 *	0.055 *	0.064 *	-
Hb difference (pre- and post-op) (mg/dL)	2.9 (1.6)	3.2 (1.3)	2.9 (1.7)	0.355 *	0.901 *	0.595 *	0.032 *	0.296
Clavien–Dindo classification (mean, sd)		27.3 (16.585)	26.27 (18.391)	0.762 *	0.09 *	0.038 *	0.736 *	0.604

sd: standard deviation; mL: milliliters; min: minutes; cm: centimeters; Rd: residual disease; mg/dL: milligrams per deciliter; ICU: intensive care unit; pRBCs: packed red blood cells; Hb: hemoglobulin; pre-op: preoperative; post-op: postoperative; LMJ: Ligasure Maryland Jaw open/sealer divider; SCS: Surgical Complexity Score. ^$^ percentages calculated within total study population; ^$$^ percentages calculated within LMJ group; ^$$$^ percentages calculated within non-LMJ group. * *p*-value for independent samples *t*-test. ** *p*-value for Pearson chi-square test. *** *p*-value for Mann–Whitney U-test. # Multivariate analysis presents statistical significance (*p*-value of F-statistic) of perioperative parameters (continuous variables) adjusted for surgical complexity score and residual disease.

## Data Availability

The raw data supporting the conclusions of this article will be made available by the authors on request.
